# *ngn-1*/neurogenin Activates Transcription of Multiple Terminal Selector Transcription Factors in the *Caenorhabditis elegans* Nervous System

**DOI:** 10.1534/g3.120.401126

**Published:** 2020-04-09

**Authors:** Elyse L. Christensen, Alexandra Beasley, Jessica Radchuk, Zachery E. Mielko, Elicia Preston, Sidney Stuckett, John I. Murray, Martin L. Hudson

**Affiliations:** * Department of Molecular and Cellular Biology, Kennesaw State University, GA 30144; † Perelman School of Medicine, University of Pennsylvania, Philadelphia, PA 19104

**Keywords:** cell fate specification, basic-helix-loop-helix, RNAseq, comparative transcriptome, proneural

## Abstract

Proper nervous system development is required for an organism’s survival and function. Defects in neurogenesis have been linked to neurodevelopmental disorders such as schizophrenia and autism. Understanding the gene regulatory networks that orchestrate neural development, specifically cascades of proneural transcription factors, can better elucidate which genes are most important during early neurogenesis. Neurogenins are a family of deeply conserved factors shown to be both necessary and sufficient for the development of neural subtypes. However, the immediate downstream targets of neurogenin are not well characterized. The objective of this study was to further elucidate the role of *ngn-1*/neurogenin in nervous system development and to identify its downstream transcriptional targets, using the nematode *Caenorhabditis elegans* as a model for this work. We found that *ngn-1* is required for axon outgrowth, nerve ring architecture, and neuronal cell fate specification. We also showed that *ngn-1* may have roles in neuroblast migration and epithelial integrity during embryonic development. Using RNA sequencing and comparative transcriptome analysis, we identified eight transcription factors (*hlh-34*/NPAS1, *unc-42*/PROP1, *ceh-17*/PHOX2A, *lim-4*/LHX6, *fax-1*/NR2E3, *lin-11*/LHX1, *tlp-1*/ZNF503, and *nhr-23*/RORB) whose transcription is activated, either directly or indirectly, by *ngn-1*. Our results show that *ngn-1* has a role in transcribing known terminal regulators that establish and maintain cell fate of differentiated neural subtypes and confirms that *ngn-1* functions as a proneural transcription factor in *C. elegans* neurogenesis.

Defects during nervous system development are implicated in numerous neurological diseases that are polygenic in nature, such as autism spectrum disorder (ASD) and schizophrenia (Ho *et al.* 2008; Xiao *et al.* 2014; Happé *et al.* 2006; Stachowiak *et al.* 2013; Zhou *et al.* 2016). In addition, genome-wide association studies indicate that polymorphisms linked to ASD and schizophrenia often map to non-coding regions of the genome housing control elements for gene transcription (Freitag 2007; Ripke *et al*. 2011). These findings suggest that some neurodevelopmental disorders may manifest as the result of changes in gene regulation.

Some of the earliest acting regulators of neurogenesis are the neurogenin genes, which code for a family of basic-helix-loop-helix (bHLH) transcription factors (Yuan and Hassan 2014; Guillemot and Hassan 2017). These proteins form obligate heterodimers via interactions between their helix-loop-helix domains (Figure 1A). These heterodimers then bind to *cis*-regulatory regions of other genes via their “basic” domains, which contain multiple conserved basic amino acids that recognize the canonical E-box motif “CANNTG” (Figure 1B). This interaction either enhances or suppresses transcription of downstream target genes, although the identity of such targets is not well characterized (Massari and Murre 2000; Grove *et al.* 2009; Waterhouse *et al.* 2009).

**Figure 1:**
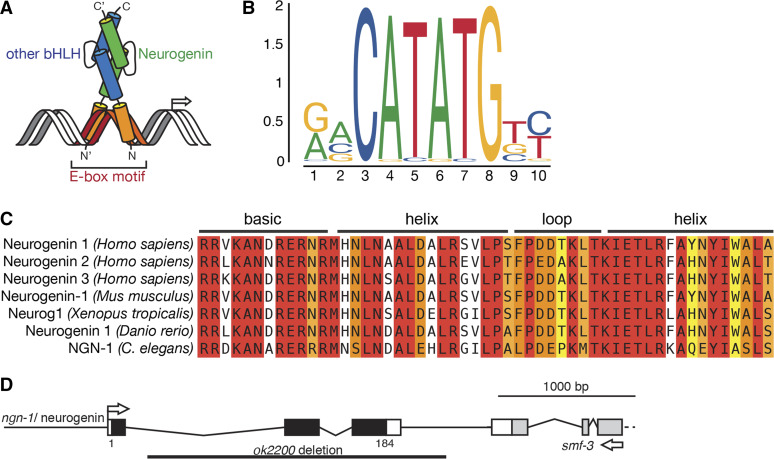
Overview of NGN-1/neurogenin structure, binding specificity, and sequence homology. (A) Schematic of NGN-1/neurogenin dimerization and DNA binding interactions. NGN-1 is a basic-helix-loop-helix (bHLH) transcription factor and is predicted to heterodimerize with another bHLH binding partner via its conserved helix-loop-helix domains (green and blue), while interacting with DNA via the basic domains (orange). (B) Illustration of the human neurogenin 1 preferential binding motif downloaded from the JASPAR database (http://jaspar.genereg.net/matrix/MA0623.2/). This conforms to a classic E-box “CANNTG” sequence. (C) Sequence homology of human, mouse, *Xenopus*, Zebrafish, and *C. elegans* NGN-1 neurogenins. Amino acids in red are 100% conserved while amino acids in orange and yellow are structurally similar. Alignment created using Jalview. (D) Diagram of the *ngn-1* locus showing the size and approximate breakpoints of the *ok2200* deletion allele. The 3′ end of adjacent gene *smf-3* is included for context and to demonstrate that the *ok2200* allele is unlikely to interfere with *smf-3* gene function. Figure 1A adapted from Roschger and Cabrele (2017). Figure 1B adapted from Fornes *et al.* (2020).

Mammals have three neurogenin genes with neurogenin 1 and 2 active during neurogenesis, and a third (neurogenin 3) required for the development of pancreatic tissue and insulin-secreting cells (Sommer *et al.* 1996; Ma *et al.* 1998; Ma *et al.* 1999; Gu *et al.* 2002; Seo *et al.* 2007). Neurogenin 1 promotes neural development but actively inhibits astrocyte differentiation (Sun *et al.* 2001). In addition, homozygous neurogenin 2 null mutant mice have developmental defects in the forebrain, dorsal root ganglia, and distal cranial ganglia (Fode *et al.* 1998; Hand and Polleux 2011). These animals also exhibit defective axon targeting in the corpus callosum, aberrant projections across the midline, and defasciculation of axon bundles. Finally, injection of mouse neurogenin mRNA into *Xenopus* embryos caused ectopic neurogenesis in ectodermal tissue (Ma *et al.* 1996). Taken together, these data suggest a critical role for neurogenins in the regulation of neurogenesis and suggests that neurogenins likely function near the top of a proneural fate specification cascade.

In humans, neurogenin 1 is associated with multiple neurodevelopmental disorders. Two single nucleotide polymorphisms associated with schizophrenia fall within the regulatory region of the neurogenin 1 locus (Fanous *et al.* 2007). In addition, a patient with congenital cranial dysinnervation disorder, a neurological condition characterized by facial paralysis including hearing loss and lack of facial expression, was found to have a homozygous deletion of the neurogenin 1 gene (Schröder *et al.* 2013). In another study, patients with homozygous mutations in neurogenin 3 were diagnosed with hypogonadotropic hypogonadism, a disorder characterized by improper function of the pituitary gland. (Germán-Díaz *et al.* 2017; Rubio-Cabezas *et al.* 2011; Rubio-Cabezas *et al.* 2016; Sayar *et al.* 2013).

The association of neurogenins with multiple human neurological disorders demonstrates the need for greater understanding of neurogenin function during nervous system development. Neurogenins are highly conserved across phyla (Figure 1C). The nematode *Caenorhabditis elegans* contains a single neurogenin ortholog, *ngn-1*, making this a useful model organism to further investigate this gene’s role in nervous system development. Forward genetic screens identified a role for *ngn-1* in defining the fate of the MI pharyngeal motorneuron (Nakano *et al.* 2010). Despite this, little is known about downstream regulatory targets of *ngn-1*. The aim of our study was to further characterize *ngn-1**’s* role in nervous system development by taking a candidate gene approach coupled with comparative transcriptomics and genetic validation. We found that *ngn-1* is required for embryonic development, has roles in establishing nerve ring architecture, and is required for AIY interneuron axon outgrowth and guidance. We also show that *ngn-1* activates transcription of almost 100 genes including eight downstream transcription factors (*hlh-34*/NPAS1, *unc-42*/PROP1, *ceh-17*/PHOX2A, *lim-4*/LHX6, *fax-1*/NR2E3, *lin-11*/LHX1, *tlp-1*/ZNF503, and *nhr-23*/RORB). *ngn-1* also has roles in transcriptional repression, suppressing the transcription of almost 500 target genes, although its role here may be indirect and via non-cell autonomous mechanisms. These data establish *ngn-1* as a key proneural gene in *C. elegans* nervous system development and demonstrate the value of comparative transcriptomics for identifying transcription factor down-stream targets.

## Materials and Methods

### Strains and maintenance

*C. elegans* strains were grown on nematode growth medium plates (NGM Lite) at 20° as described previously (Brenner 1974). N2 (Bristol) was used as the wild-type strain. The following alleles were used in the course of this study: LGI *dpy-5**(**e907**)* and *mab-20**(**bx24**)*, LGII *vab-1**(**dx31**)*, LGIII *cnd-1**(**ju29**)*, *cnd-1**(**gk718**)* and *unc-119**(**ed3**)*, LGIV *daf-18**(**ok480**)*, *ngn-1**(**ok2200**)* and *efn-4**(**bx80**)*, LGV *him-5**(**e1490**)*, LGX *sax-3**(**ky123**)* and *sdn-1**(**zh20**)*. Integrated transgenes used in the course of this study were *juIs76** [unc-25p*::*GFP + **lin-15**(+)]*, *mgIs18** [ttx-3p*::*RFP]*, *nIs394** [ngn-1*::*GFP + **lin-15**(+)]*, *otIs33** [**kal-1**p*::*GFP]*, *sIs14542** [hlh-34p*::*GFP + **dpy-5**(+)]*, and *ujIs113** [pie-1*::*mCherry*::*Histone H2B* + *nhr-2p*::*mCherry*::*HIS-24* + *unc-119**(+)]*. Extrachromosomal arrays used in this study were *leEx1829 [unc-42p*::*GFP* + *unc-119**(+)]*, *quEx99** [**sax-3**(minigene) + odr-1p*::*RFP]*, and *wwEx37** [ngn-1p*::*GFP + **unc-119**(+)]*. The chromosomal rearrangement *mIn1** [**mIs14* *dpy-10**(**e128**)]* II was used to balance a lethal construct. All mutants were outcrossed at least four times except *cnd-1**(**gk718**)* (2x), *vab-1**(**dx31**)* (1x), *daf-18**(**ok480**)* (2x), and *sax-3**(**ky123**)* (3x). Double and triple mutant strains bearing reporter genes were constructed using standard genetic techniques. See Table S1 for details of strains generated during the course of this study including strain numbers and extra-chromosomal array construction.

### Microscopy

Well-fed worms grown under normal conditions were used to characterize expression patterns. Images were captured using either a Zeiss LSM 700 confocal microscope with Zen Black imaging software, a Zeiss AxioImager Z2 fluorescent microscope with Zen Blue imaging software, or an Olympus BX61 fluorescence microscope with CellSenseDimension software. Adult worms were staged for imaging by transferring 10-20 L4 hermaphrodites to a new plate and imaging those worms 24 hr later. Worms were mounted on 2% agarose pads, anesthetized with 3.5 μl of 10 mg/ml sodium azide solution, and then immobilized under an 18 × 18mm cover slip. Images were analyzed using Fiji software (Rueden *et al.* 2017; Schindelin *et al.* 2012). For quantitative imaging of *leEx1829 [unc-42p*::*GFP* + *unc-119**(+)]* and *ngn-1**(**ok2200**)*; *leEx1829 [unc-42p*::*GFP* + *unc-119**(+)]* animals, the head regions of L1 larvae (collected within an hour of hatching) were captured as an image stack using a Zeiss LSM 700 confocal microscope at 40x magnification under identical settings, ensuring no detector saturation. Images were processed in Fiji using a Z-project - Sum Slices workflow, which rendered the summated stacks as 32-bit images. The head, from the nose tip to the posterior end of the pharynx was outlined using a segmented line and the region outside the line deleted using the Edit – Clear Outside function. Pixel values and counts were obtained using the Analyze - Histogram function (using the pixel value range) then processed in Microsoft Excel (sum [pixel value x pixel count]) and expressed as arbitrary fluorescence units.

### Lethality assays

L4 hermaphrodites were transferred to NGM Lite plates, one worm per plate. After 24 hr, each hermaphrodite was transferred to a new plate. 24 hr after the removal of the hermaphrodite, each plate was scored for dead embryos and live L1/L2 larvae. The plates were rescreened 48 hr later for live adults, and the number of adults was subtracted from the L1/L2 larval counts to determine larval lethality. Adults were transferred until they died or until no more viable embryos were observed. Dead embryo and larvae counts were compared to wild-type worms and statistical analysis was performed using Fisher’s exact test.

### Embryo laying assay

Healthy well-fed young adults were transferred to plates containing fresh OP50 *E. coli* and allowed to lay embryos for one hour. Embryos were promptly picked to agarose pads and imaged at 40x magnification on an Olympus BX61 microscope equipped with DIC optics. Developmental timing was determined by counting the nuclei visible on the uppermost surface. Embryos with greater than ∼30 nuclei visible were timed by counting nuclei along the anterior-posterior and left-right midlines, and multiplying the two values.

### 4-dimensional time-lapse microscopy of embryonic development

Two to four cell embryos were obtained by picking young adult hermaphrodites into a watch glass filled with M9 buffer then cutting each worm at the vulva using a number 10 blade scalpel. Embryos were transferred to a freshly made 2% agarose pad then sealed under a coverslip using a small drop of immersion oil at each corner.

High resolution differential interference contrast (DIC) z-stack images (1 µm steps, 27–30 slices) were collected using a 63x magnification oil immersion objective on a Zeiss AxioImager Z2 microscope equipped with motorized z-axis stage. Data stacks were captured every 2 min for 300 cycles using Zen Blue software and analyzed using Fiji. The following developmental time points were scored: Ea/Ep cell ingression, gastrulation cleft opening, gastrulation cleft closure, ventral enclosure, comma stage, and elongation. Scores for mutant strains were compared to wild-type worms and statistical analysis was performed using the student’s *t*-test.

### kal-1p::GFP cell lineage analysis

A *kal-1p*::*GFP* lineaging strain was constructed by crossing *otIs33** [kal-1p*::*GFP]* with a mCherry histone marker line, *ujIs113** [pie-1*::*mCherry*::*Histone H2B* + *nhr-2p*::*mCherry*::*HIS-24 + **unc-119**(+)]*. Embryo preparation and microscopy were performed according to previous studies (Richards *et al.* 2013). Briefly, early embryos up to the four-cell stage were mounted onto microscope slides using the bead method (Bao and Murray 2011). 4D time-lapse images with a scan field dimension of 712×512×67 pixels (0.087×0.087×0.504 μm) were taken every minute using an inverted Leica SP5 TCS Resonance-scanning confocal microscope. A custom stage insert (Brook Industries, LakeVilla, IL) maintained temperature control of the embryos during imaging. Automated cell lineage tracing was performed for 240 time points using StarryNite (Bao *et al.* 2006) and curation of the cell lineage was done using AceTree (Boyle *et al.* 2006).

### RNA extraction and sequencing

Wild-type and *ngn-1**(**ok2200**)* mutants and were grown in liquid culture in M9 buffer supplemented with 1 mg/ml cholesterol, and 5 mls of 50% OP50 *E. coli* slurry as described previously (Hudson *et al.* 2006). Embryos were isolated by pelleting worms and resuspending in 1.2% NaOCl in 0.5M NaOH until only embryos remained, then washed three times and resuspended in M9 buffer. 200-300 μl of embryos were added to a mortar containing liquid N_2_ and ground to a fine powder then total RNA extracted using RiboZol (RIBOSOL) according to the vendor’s protocol and the aqueous phase separated by centrifugation in MaXtract High Density tubes (QIAGEN, Germantown, MD). The aqueous phase was precipitated using an equal volume of 100% isopropanol plus 3 μl of 20 ng/ml glycogen, the RNA pellet washed once in isopropanol, once in 70% ethanol then resuspended in 50μl of diethylpyrocarbonate (DEPC) treated water. RNA concentration was estimated using a Thermo Scientific NanoDropTM Lite. Samples were then diluted to less than 10 ng/μl and analyzed on an Agilent 4200 Tapestation using High Sensitivity RNA ScreenTape. Only samples with an RNA integrity number (RIN) number of 8.9 or greater were sequenced. Four biological replicates of wild-type and *ngn-1**(**ok2200**)* embryonic total RNA were sent to the University of Kansas Genome Sequencing Core for sequencing via an Illumina NextSeq 550 system (high output, single read 150bp sequencing).

### RNA alignment and analysis of RNA sequencing data

RNA sequencing data analysis was performed using the Galaxy platform (Afgan *et al.* 2018). RNA-seq read quality was determined using FastQC ver.0.11.7. Read files were converted to sanger files and groomed for read quality using FASTQ Groomer ver.1.1.1. Reads were aligned back to the *C. elegans* reference genome (wbcel235/ce11) using Hisat2 ver.2.1.0 with Samtools 1.4. Aligned reads were assembled and transcripts quantified using Stringtie 1.3.3.2 via Samtools 1.6. Differential expression analysis was performed using DESeq2 ver.3.4.1 (galaxy.org ver.2.11.40.2). Tissue Enrichment and Gene Ontology analyses were performed using a web-based implementation of the Angeles-Albores *et al.* (2016) tools available at https://www.wormbase.org/tools/enrichment/tea/tea.cgi.

### Data availability

Reagents generated in this study are available on request. Supplementary tables and figures are available via the GSA Figshare portal. Figure S1 shows the precocious embryo laying phenotype of *ngn-1**(**ok2200**)* mutants. Figure S2 shows a representative *kal-1p*::*GFP* cell lineage. Figure S3 shows *ngn-1*; *guidance cue* double-mutant analysis of AIY neuron axon outgrowth. Table S1 shows strains generated in this study. Table S2 shows the inferred terminal cell fates identified in the *kal-1p*::*GFP* cell lineage. Tables S3 and S4 show the up-regulated and down-regulated lists of *ngn-1* transcriptome hits identified in this work. Transcriptome files generated in this study are publicly available via the Gene Expression Omnibus (Edgar *et al.* 2002), accession number GSE143599 (https://www.ncbi.nlm.nih.gov/geo/query/acc.cgi?acc=GSE143599). Supplemental material available at figshare: https://doi.org/10.25387/g3.12034392.

## Results

### ngn-1 is required for embryonic viability and neuromuscular development

**Figure 2:**
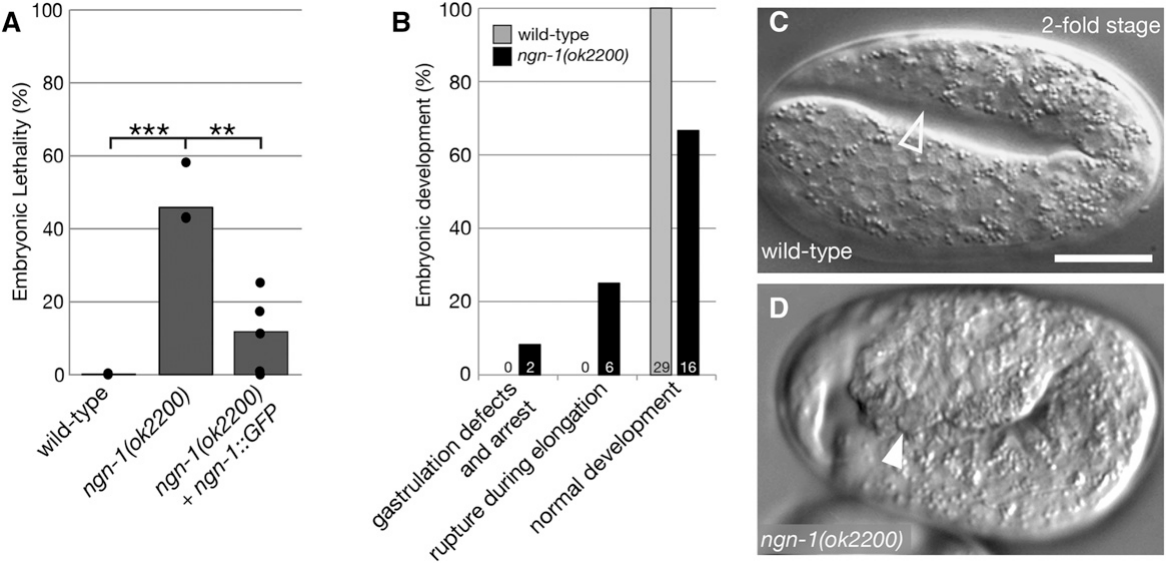
*ngn-1* is required for normal embryogenesis. (A) Percentage embryonic lethality in wild type, *ngn-1**(**ok2200**)*, and *ngn-1**(**ok2200**)* + rescuing *ngn-1*::*GFP* strains. n = 4, 3, and 5 whole broods respectively. Black circles show individual broods. ** *P* < 0.01, *** *P* < 0.001, student *t*-test plus Bonferroni correction for multiple comparisons. (B) Classification of embryonic arrest phenotypes in wild type and *ngn-1**(**ok2200**)* mutant embryos as assayed by 4D-time-lapse video microscopy. n = 29 and 24 broods respectively. (C, D) Representative images of wild type and *ngn-1**(**ok2200**)* mutants at the twofold stage of embryogenesis. (C) Open arrowhead shows the ventral region toward the tail of the animal. (D) Filled arrowhead shows internal cells rupturing from the same region in an *ngn-1* mutant. Scale bar in panel C = 10 μm.

Beyond the role of *ngn-1* in defining MI pharyngeal motorneuron cell fate, little is known about this deeply conserved gene in *C. elegans* nervous system development (Nakano *et al.* 2010). To further characterize *ngn-1*, we examined worms carrying the *ok2200* mutant allele, which is a 2266bp deletion/7 bp insertion in the *ngn-1* locus and predicted to be a null mutation (Figure 1D). In our study, *ngn-1**(**ok2200**)* mutants showed 45% embryonic lethality (Figure 2A, Table 1). *ngn-1**(**ok2200**)* animals bearing a *ngn-1*::*GFP* translational transgene (Nakano *et al.* 2010) are rescued for embryonic developmental defects, showing significantly less embryonic lethality (10% compared to 45% in *ok2200* mutants alone, *P* < 0.01), indicating that the embryonic lethal phenotype is caused by the *ngn-1**(**ok2200**)* allele and not a closely linked second-site mutation. *ngn-1* mutant animals that survived to adulthood showed sluggish, uncoordinated movement, aberrant head movement, and precocious embryo laying (Supplemental Figure 1). Overall, these observations suggest that *ngn-1* is required for a broad array of neuromuscular functions.

**Table 1:** Summary of embryonic and larval lethal interactions between *ngn-1(ok2200)*, *cnd-1* and canonical axon guidance and cell migration mutants. *P < 0.005; **P < 0.001; ***P < 0.0001, 2-tailed student t-test and Bonferroni correction for multiple comparisons. Note that double mutants were compared against the single mutant that showed the strongest phenotype. *ngn-1*; *vab-1* and *ngn-1*; *sax-3* double mutants could not be isolated away from balancer chromosomes or rescuing transgenes suggesting 100% embryonic lethality in the unbalanced strains

Genotype	Embryonic Lethality (SD)	Larval Lethality (SD)	Broods	N
wild-type	0.11 (0.2)	0.12 (0.2)	4	823
*ngn-1(ok2200)*	45.9 (4.8) ***	10.2 (2.2) *	3	482
*ngn-1*; *ngn-1*::*GFP*	9.8 (10.6) ***	ND	5	471
*cnd-1(ju29)*	1.5 (1.4)	1.0 (1.2)	4	783
*ngn-1*; *cnd-1*	45.5 (6.1)	26.1 (5.2)	8	1967
*cnd-1(gk718)*	7.9 (1.4)	12.4 (2.0)	6	1464
*ngn-1*; *cnd-1(gk718)*	51.7 (5.5)	18.7 (1.1) **	6	851
*daf-18(ok480)*	3.3 (1.0)	0.3 (0.5)	6	1586
*ngn-1 daf-18(ok480)*	64.3 (5.4)	17.1 (5.6)	4	715
*sdn-1(zh20)*	7.7 (5.3)	5.5 (3.7)	5	868
*ngn-1*; *sdn-1(zh20)*	11.8 (8.5) *	10.4 (5.9)	5	656
*efn-4(bx80)*	14.3 (2.5)	8.0 (3.3)	6	927
*ngn-1 efn-4(bx80)*	60.1(15.3)	13.1(1.9)	3	389
*mab-20(bx24)*	15.2 (7.2)	5.0 (4.1)	5	905
*ngn-1 mab-20(bx24)*	32.3 (7.3)	3.1 (1.0) *	5	1510
*vab-1(dx31)*	6.7 (5.0)	18.4 (4.1)	5	763
*ngn-1*; *vab-1(dx31)*	ND	ND		
*sax-3 (ky123)*	70.1 (17.2)	10.3 (8.2)	3	635
*ngn-1*; *sax-3(ky123)*	ND	ND		

To further investigate the role of *ngn-1* in embryonic development, we scored whole broods of *ngn-1* mutants for embryonic lethality and larvae that died before reaching adulthood (Table 1). In addition to the 45% embryonic lethality there was 10% larval lethality suggesting that *ngn-1* is required for one or more processes essential to larval health (Table 1, 10.2% larval lethal, *P* < 0.005).

To determine when *ngn-1* mutants were dying during embryonic development, we used 4-dimensional (4D) time-lapse microscopy to observe developing *ngn-1**(**ok2200**)* embryos from the 2-4 cell stage through just prior to hatching (10 hr of development, Figure 2B - D). Surprisingly, only 8% of *ngn-1* mutant embryos (2/24 observed) showed defects in gastrulation consistent with errors in neuroblast migration when compared to wild-type embryos (0/29 observed). Instead, the main arrest stage observed (25% of embryos) was due to a posterior ventral rupture during elongation (6/24 *ngn-1**(**ok2200**)* embryos compared to 0/29 wild type). While only 33% of *ngn-1**(**ok2200**)* embryos arrested during 4D imaging when compared to 46% when measured by whole brood analysis, this discrepancy is likely a result of the small sample size (n = 24) used in this assay. These data suggest that *ngn-1* plays only a minor role in neuroblast migration but may have a non-cell autonomous role in posterior epithelial integrity.

### ngn-1 is required for organization of the nerve ring and neuronal cell body location

**Figure 3:**
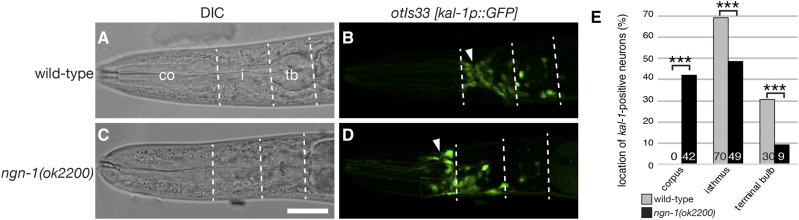
*ngn-1* is required for normal development of the nerve ring and anterior nervous system. (A - D) DIC images and parallel *kal-1p*::*GFP* expression in wild type (A, B) and *ngn-1**(**ok2200**)* (C, D) mutants respectively. Dashed lines show the boundaries between the corpus (co), isthmus (i), and terminal bulb (tb) respectively and were used to score the location of *kal-1p*::*GFP* expressing cells. Filled arrowheads in B and D show the location of the nerve ring. Scale bar in panel C = 20 μm. (E) Location of *kal-1p*::*GFP* expressing cells in wild type (gray) and *ngn-1* mutants (black) respectively. ***, *P* < 0.001, student *t*-test.

To further understand the role of *ngn-1* in development of the nervous system, *ngn-1**(**ok2200**)* mutants were crossed with a *kal-1p*::*GFP* reporter gene, which labels a subset of neurons and glia in the head region (Supplemental Figure 2; Supplemental Table 2; Bülow *et al.* 2002). In wild type animals bearing the *kal-1p*::*GFP* reporter gene, the nerve ring (a tight bundle of neuronal processes where many of the worm’s synaptic connections are found) appears as a distinct narrowing of fluorescence around the isthmus of the pharynx (arrowhead, Figure 3A, B). In *ngn-1* mutants, this nerve ring architecture is completely lost (arrowhead, Figure 3C, D). In addition, numerous cell bodies were anteriorly displaced and showed aberrant dendritic projections (Figure 3D). We characterized neuronal cell body displacement by counting the number of *kal-1p*::*GFP*-positive cell bodies in three regions of the head: the corpus, the isthmus, and the terminal bulb. In wild-type animals, an average of 18.5 *kal-1p*::*GFP*-positive neurons were visible in the head region (n = 9 worms scored) and 12.8 cell bodies (70%) were located in the isthmus region, with the remaining 5.7 (30%) clustered around the terminal bulb (Figure 3E). *ngn-1* mutants showed around the same number of *kal-1p*::*GFP*-positive head neurons (17.8 average, n = 7 worms scored). However, many neurons were anteriorly displaced, with an average of 7.5 neurons (42%) now located in the corpus region (*P* < 0.001), 8.7 neurons in the isthmus (42%, *P* < 0.001) and only 1.7 neurons (9%, *P* < 0.001) in line with the terminal bulb. These data indicate the *ngn-1* has roles in controlling both nerve ring architecture and neuronal cell body positioning.

### ngn-1 controls AIY neuron axon outgrowth, polarity and cell fate

**Figure 4:**
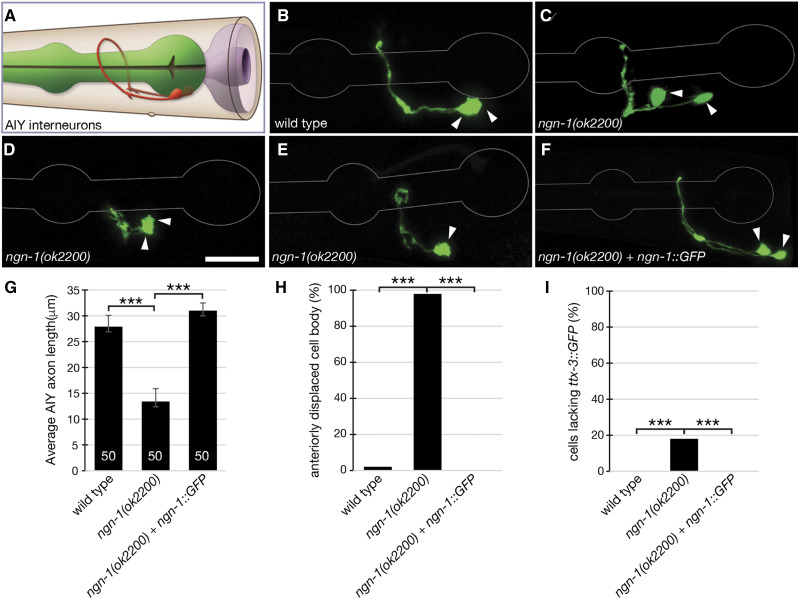
*ngn-1* is required for accurate cell body positioning, axon outgrowth and cell fate specification in AIY interneurons. (A) Schematic showing AIYL and R interneuron cell body positioning and axon location (image adapted from www.wormatlas.org). (B – F). Confocal micrographs of *ttx-3p*::*GFP* expression in (B) wild type, (C - E) *ngn-1**(**ok2200**)* mutants, and (F) *ngn-1**(**ok2200**)* mutants rescued with an *ngn-1*::*GFP* transgene (note that *ngn-1*::*GFP* is not visible in young adults). Arrowheads show AIYL/R cell body location. These are anteriorly displaced in *ngn-1* mutants. (D) AIYL/R axons fail to extend to the dorsal side. (E) Only a single AIY neuron is present. (G – I) Summary of wild type and *ngn-1**(**ok2200**)* axon outgrowth and AIY fate specification phenotypes. n = 50 worms analyzed for each strain. ***, *P* < 0.001, student *t*-test plus Bonferroni correction for multiple comparisons. Scale bar in panel D = 10 μm.

To gain a better understanding of *ngn-1* function at the single cell level, we used a *ttx-3p*::*GFP* reporter gene to examine axon outgrowth in the AIY interneurons (Hobert *et al.* 1997). In wild type animals, the AIY left and right cell bodies typically lie below and just posterior to the pharyngeal terminal bulb and extend anterior processes that meet under the pharynx then enter the nerve ring, extending toward the dorsal side where they again make contact via a gap junction (Figure 4A and 4B) (White *et al.* 1986). In *ngn-1* mutants, 98% of animals (49/50) show anteriorly displaced AIY cell bodies (Figure 4C and 4H). In addition, 100% of *ngn-1* mutants (50/50, *P* < 0.001) showed defective dorsal axon extension, such that the AIYL and R axons fail to meet on the dorsal side (Figure 4C – E). The average AIY axon length in *ngn-1* mutants was 12.3 μm (AIYL, n = 28) and 10.2 μm (AIYR, n = 34) compared with 28 μm (AIYL and R, n = 50 each) in wild type (Figure 4G). Some *ngn-1* mutants (19% of neurons scored, 17/89) showed such severe outgrowth defects that the axons remained below the pharynx, appearing as a tangle (Figure 4D). We also observed loss of *ttx-3p*::*GFP* expression in 16% of *ngn-1* mutants examined, resulting in only a single GFP-positive cell being apparent (Figure 4E and 4I). *ngn-1**(**ok2200**)* animals carrying the *ngn-1*::*GFP* translational reporter gene showed complete rescue of cell body displacement and axon outgrowth defects, further confirming that the phenotypes observed were due to *ngn-1* loss-of-function and not a closely-linked mutation (Figure 4F – H). Overall, these data indicate that *ngn-1* is required for multiple neural developmental functions including cell body positioning, axon outgrowth, and axon guidance. In addition, *ngn-1* has a partly redundant role in driving *ttx-3* expression, which is the terminal selector gene for AIY fate, but likely functions in parallel with one or more additional genes in this process.

### ngn-1 has no obvious role in controlling canonical neuroblast migration or axon guidance genes

Considering the axon guidance phenotypes seen in *ngn-1* null mutants, it is likely that *ngn-1* controls the transcription of multiple genes required for neural development. We took a candidate gene approach to identifying regulatory targets of *ngn-1* based on the phenotypes observed in *ngn-1**(**ok2200**)* mutants (Table 1). Many of these genes play a role in neuroblast migration or axon guidance during embryonic development, and loss-of-function mutations typically exhibit mild to strong embryonic lethality as a result of defects in these processes (George *et al.* 1998; Zallen *et al.* 1998; Chin-Sang *et al.* 2002; Hudson *et al.* 2006). We generated *ngn-1*; *candidate gene* double mutants and compared embryonic and larval lethality against each single mutant. Overall, the majority of the double mutant strains showed, at best, additive but not significant increases in embryonic lethality when compared to *ngn-1* alone (Table 1), suggesting that *ngn-1* does not exhibit strong genetic interactions with these known axon guidance cues.

To further examine the genetic interactions between candidate axon guidance genes and *ngn-1* during axon outgrowth, *ngn-1*; *candidate gene* double mutants were assayed for axon outgrowth and guidance defects in the AIY interneurons (Wang *et al.* 2008; Christensen *et al.* 2011; Hartin *et al.* 2015; Schwieterman *et al.* 2016). Each double mutant showed similar average AIY axon lengths when compared to *ngn-1**(**ok2200**)* alone, suggesting that the *ok2200* phenotype masks defects seen in each single mutant (Supplemental Figure 3). Based on these data, it was unclear if *ngn-1* has any role in the transcriptional regulation of these axon guidance cues.

### RNAseq identifies multiple ngn-1 transcriptional targets

**Figure 5:**
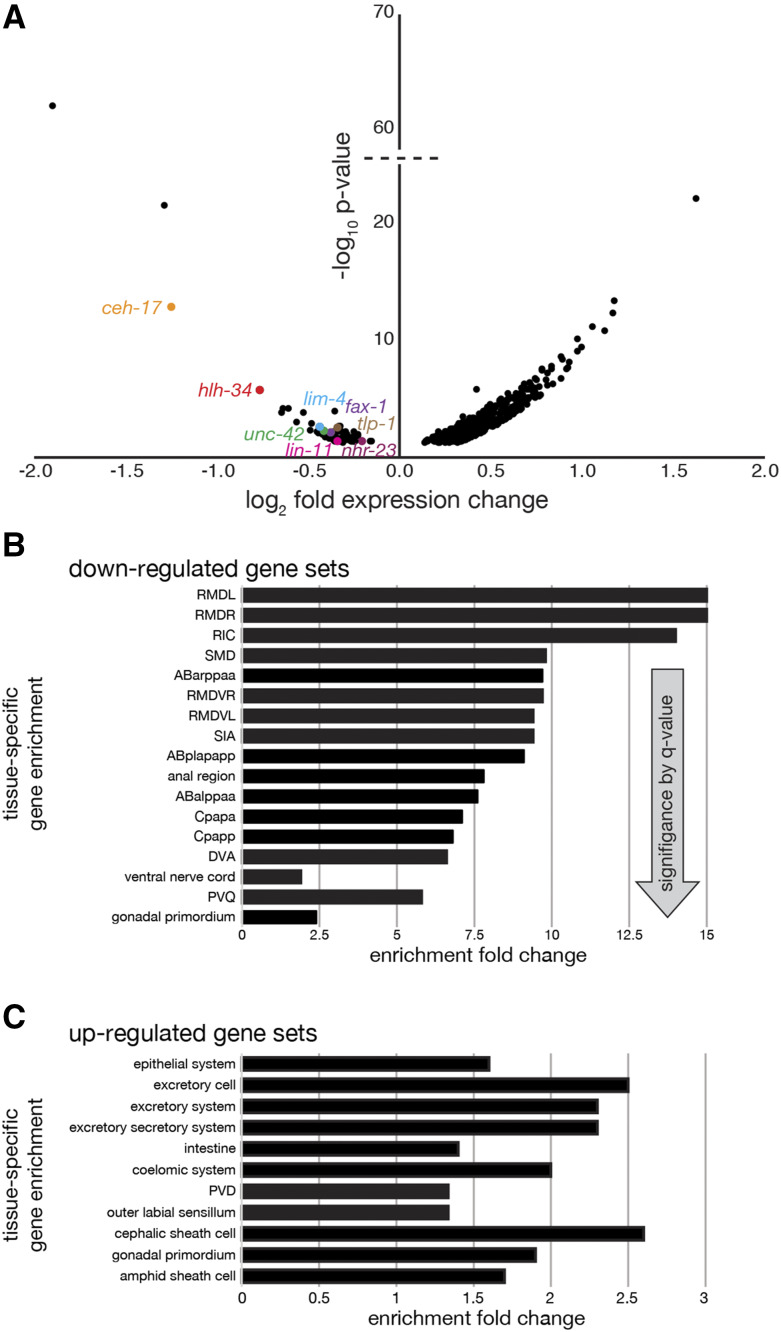
Comparative transcriptome analysis of *ngn-1**(**ok2200**)* mutants identifies multiple genes that are up- or down-regulated by *ngn-1*. (A) Volcano plot of log_2_ fold expression changes *vs.* log_10_ significance (p-value) summarizing transcript differences between wild type and *ngn-1**(**ok2200**)* mutants. Down-regulated transcription factor genes are highlighted in color. (B, C) Bioinformatic enrichment analyses of transcriptome hits. RNAseq hits were bioinformatically compared against “gold-standard” curated lists of genes previously shown to be associated with a single cell or tissue type. (B) Down-regulated gene sets primarily associate with neurons or cells derived from the pro-neural AB cell lineage. (C) Up-regulated gene sets primarily associate with the epithelial, intestine or excretory system. Arrow shows decreasing significance by q-value (hypergeometric test followed by a Benjamini-Hochberg correction for false discovery).

RNA sequencing (RNAseq) is a powerful genetic tool to both identify and quantify gene expression (Conesa *et al.* 2016; Liao *et al.* 2014; Love *et al.* 2014). As our candidate gene approach to identifying *ngn-1* regulatory targets was unsuccessful, we used RNAseq to identify transcript differences between wild type and *ngn-1**(**ok2200**)* mutant embryos. This unbiased approach was employed to characterize components of the *ngn-1* gene regulatory network downstream of *ngn-1*. To generate a comparative transcriptome of wild type and *ngn-1**(**ok2200**)* null mutants (n = 4 replicates each), RNA was isolated from mixed-stage embryos, sequenced, and analyzed. This generated a list of 587 differentially expressed genes with a p-value ≤ 0.05 (Supplemental Tables 3 and 4). We found that the majority of these genes (497/587, 85%) were upregulated in the *ngn-1* mutant background, suggesting that *ngn-1* acts primarily as a transcriptional repressor during embryogenesis (Figure 5A). When we looked at the 40 most significant hits from the differential expression analysis based on p-value, five genes were down-regulated, three of which are predicted to code for transcription factors; *ngn-1* (as expected from the comparative transcriptome of a deletion mutant), *ceh-17*, and *hlh-34* (Table 2A). Six additional transcription factor genes had significantly lower transcript levels in *ngn-1* mutants when compared to wild type (*fax-1*, *lim-4*, *lin-11*, *unc-42*, *tlp-1*, and *nhr-23*), but were outside the 40 most significant hits (Figure 5A). Conversely, the up-regulated genes were primarily of uncharacterized function (n = 15/40). Whether *ngn-1* controls the transcription of these genes directly or indirectly (via transcription of one or more intermediate factors or via non-cell autonomous mechanisms) is not known.

**Table 2:** *ngn-1* transcriptome most significant hits by p-value. (A) Down-regulated genes. (B) Up-regulated genes. A. *ngn-1* transcriptome down-regulated genes (most significant p - value) B. *ngn-1* transcriptome up-regulated genes (most significant p - value)

Gene ID	Gene Name	Base mean	log2(FC)	P-value	P-adj
WBGene00003595	*ngn-1*	283	−3.9	3.2E-130	5.2E-126
WBGene00021766	*hex-4*	850	−1.9	9.4E-63	7.8E-59
WBGene00001816	*haf-6*	254	−1.3	2.4E-22	1.0E-18
WBGene00000440	*ceh-17*	43	−1.3	1.3E-13	3.6E-10
WBGene00011327	*hlh-34*	17	−0.8	1.8E-06	0.00076
WBGene00268211	*T02H6.12*	49	1.6	5.08E-23	2.77E-19
WBGene00007097	*B0024.4*	489	1.2	3.30E-14	1.08E-10
WBGene00007131	*pals-26*	221	1.2	4.24E-13	9.89E-10
WBGene00016247	*C30B5.6*	145	1.1	5.41E-12	1.10E-08
WBGene00008739	*F13D12.3*	190	1.1	1.30E-11	2.36E-08
WBGene00014173	*ZK970.7*	340	1.0	6.45E-11	1.05E-07
WBGene00011446	*T04F8.8*	79	1.0	3.85E-10	5.72E-07
WBGene00013481	*Y69H2.3*	456	1.0	8.34E-10	1.14E-06
WBGene00018725	*kreg-1*	77	0.9	2.05E-09	2.58E-06
WBGene00018729	*F53A9.6*	46	0.9	4.34E-09	5.06E-06
WBGene00010135	*F55H12.4*	288	0.9	6.43E-09	7.00E-06
WBGene00012185	*W01F3.2*	111	0.8	1.51E-08	1.55E-05
WBGene00017127	*E04F6.8*	75	0.9	2.10E-08	2.02E-05
WBGene00017506	*F16B4.4*	206	0.8	2.37E-08	2.07E-05
WBGene00007454	*C08F11.7*	20	0.9	2.41E-08	2.07E-05
WBGene00008301	*pals-39*	33	0.8	2.74E-08	2.24E-05
WBGene00012961	*Y47H10A.5*	178	0.8	3.00E-08	2.33E-05
WBGene00018730	*F53A9.7*	48	0.8	6.17E-08	4.59E-05
WBGene00021977	*Y58A7A.3*	226	0.9	1.53E-07	1.09E-04
WBGene00012593	*nspe-7*	69	0.7	1.63E-07	0.00011
WBGene00011772	*T14G8.4*	34	0.8	1.96E-07	0.00013
WBGene00004222	*ptr-8*	127	0.8	2.29E-07	0.00014
WBGene00138721	*pals-37*	30	0.7	4.25E-07	0.00026
WBGene00009130	*F25H5.8*	30	0.7	4.43E-07	0.00026
WBGene00007132	*pals-27*	102	0.8	5.22E-07	2.94E-04
WBGene00022231	*tyr-6*	166	0.8	6.01E-07	0.00032
WBGene00016788	*C49G7.10*	51	0.7	6.13E-07	0.00032
WBGene00044900	*cnc-11*	39	0.7	6.71E-07	0.00034
WBGene00015046	*nlp-34*	158	0.8	8.23E-07	0.00041
WBGene00003765	*nlp-27*	66	0.7	1.01E-06	0.00049
WBGene00016147	*cyp-32A1*	83	0.7	1.06E-06	0.00050
WBGene00013489	*col-42*	126	0.7	1.29E-06	0.00059
WBGene00010491	*K02B7.3*	1394	0.4	1.40E-06	0.00062
WBGene00000560	*cnc-6*	70	0.7	1.65E-06	0.00071
WBGene00007506	*C10C5.2*	31	0.7	2.10E-06	0.00086

Our RNAseq assay generated an unbiased list of all *C. elegans* genes that are differentially regulated in *ngn-1* mutants compared with wild type. We employed tissue enrichment analysis to clarify which tissues and cells our transcriptome hits associated with. To this end, we bioinformatically compared our transcriptome results against “gold-standard” curated lists of genes associated with single cell or tissue types (Angeles-Albores *et al.* 2016; Angeles-Albores *et al.* 2018). These reference datasets were generated by Fluorescence-Activated Cell Sorting of cells isolated from worms bearing cell or tissue-specific fluorescent labels, followed by RNAseq or microarray assay to determine gene expression profiles within those cell/tissue types. The bulk of our down-regulated targets identified by RNAseq were found to be associated with individual neuron subtypes such as the SIA neurons or proneural cell lineages derived from the AB founder cells, further supporting our data indicating that *ngn-1* activates or enhances transcription primarily in neuronal tissue (Figure 5B). The SIA neurons play a key role in pioneering the architecture of the nerve ring suggesting that *ngn-1* dependent transcription may have a role in defining SIA fate and/or function (Rapti *et al.* 2017). In contrast, the genes up-regulated in our *ngn-1* transcriptome were significantly enriched in the excretory, intestine, and epithelial-associated genes (Figure 5C). This raises the possibility that *ngn-1* may repress the transcription of non-neural genes in neural tissues. Alternatively, *ngn-1* may suppress transcription in non-neuronal tissues via non-cell autonomous mechanism(s).

We corroborated our tissue enrichment analysis by performing a gene ontology (GO) term enrichment analysis on our up- and down-regulated target genes (Table 3). Most of the terms enriched in the down-regulated dataset were related to transcription and DNA binding, consistent with *ngn-1* having a role in activating gene transcription. Conversely, genes in the up-regulated dataset were mostly associated with cuticle and innate immune response terms. This suggests that *ngn-1* may suppress transcription of target genes associated with the hypodermis including the innate immune response, possibly via non-cell autonomous mechanism(s).

**Table 3:** *ngn-1* transcriptome gene ontology (GO) term most significant hits by p-value. (A) GO-terms associated with down-regulated genes. (B) GO-terms associated with up-regulated genes. A. *ngn-1* transcriptome Gene Ontology analysis: down-regulated genes (most significant p - value) B. *ngn-1* transcriptome Gene Ontology analysis: up-regulated genes (most significant p - value)

Gene Ontology Term	GO-term ID	Expected	Observed	Enrichment (FC)	P-value	P-adj
iron binding	GO:0005506	0.6	5	8.4	3.3E-05	0.0042
tetrapyrrole binding	GO:0046906	0.77	4	5.2	0.0011	0.069
RNA polymerase II regulatory region DNA binding	GO:0001012	0.77	4	5.2	0.0011	0.069
transcription reg. region sequence-specific DNA binding	GO:0000976	0.84	4	4.8	0.0017	0.069
organic acid metabolic process	GO:0006082	2.2	7	3.1	0.002	0.069
neurogenesis	GO:0022008	1.4	5	3.7	0.0026	0.069
regulatory region nucleic acid binding	GO:0001067	1	4	3.9	0.0039	0.07
double-stranded DNA binding	GO:0003690	1.1	4	3.7	0.0048	0.074
cellular developmental process	GO:0048869	3.9	9	2.3	0.0062	0.086
immune system process	GO:0002376	3.5	48	14	2.1E-40	2.6E-38
response to biotic stimulus	GO:0009607	2.8	33	12	3.1E-26	1.9E-24
extracellular region	GO:0005576	8.2	43	5.3	3.6E-19	1.5E-17
extracellular space	GO:0005615	4.4	30	6.9	3.1E-17	9.7E-16
metalloendopeptidase activity	GO:0004222	1.5	10	6.6	4.1E-07	1.0E-05
membrane	GO:0016020	97	137	1.4	1.7E-05	0.00035
peptidase activity	GO:0008233	6.7	18	2.7	5.5E-05	0.00097
neuropeptide signaling pathway	GO:0007218	1.8	8	4.3	0.00011	0.0017
intrinsic component of membrane	GO:0031224	85	118	1.4	0.00018	0.0026
cation binding	GO:0043169	24	38	1.6	0.0022	0.027
incorrect protein topology response	GO:0035966	2.8	8	2.8	0.0023	0.027
zinc ion binding	GO:0008270	7.8	16	2.1	0.0025	0.027
lytic vacuole	GO:0000323	1.4	5	3.5	0.0033	0.032
molting cycle	GO:0042303	1.6	5	3.1	0.0062	0.055
structural constituent of cuticle	GO:0042302	2.4	6	2.5	0.011	0.091
protein catabolic process	GO:0030163	4.9	10	2	0.011	0.091
collagen trimer	GO:0005581	2.4	6	2.5	0.012	0.091

**Figure 6:**
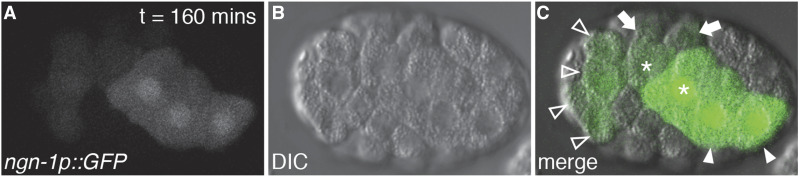
*ngn-1p*::*GFP* is expressed in AB and MS lineages during early embryogenesis. (A) *ngn-1-GFP* expression pattern 160 min post-first cell division. (B) Parallel DIC image. (C) Panel merge. Arrowheads, ABpra/p cells; asterisks, MSpa/p cells; arrows, MSaa/p cells; open arrowheads, ABar descendants.

To help clarify our understanding of how *ngn-1* might be regulating transcription at the cellular level, we performed 4D-timelapse video analysis of *ngn-1p*::*GFP* expression in the early embryo (Figure 6). We found strong *ngn-1p*::*GFP* expression in the ABpra/p cells (arrowheads, Figure 6C), along with weaker expression in ABar descendants (open arrowheads, Figure 6C). However, we also found strong expression in the MSpa/p cells (asterisks, Figure 6C) with weaker expression in MSaa/p (arrows, Figure 6C). The ABar and ABpr founder cells primarily give rise to neurons, consistent with *ngn-1’s* association with CNS-specific tissue enrichment datasets (Sulston *et al.* 1983). Also, *ngn-1* expression in the ABar lineage correlates with its role in defining the MI pharyngeal motor neuron fate (ABaraappaaa) (Nakano *et al.* 2010). *ngn-1*’s role in the MS lineage is not known. However, pharyngeal neurons, muscles and accessory gland cells are derived from the MS lineage, suggesting that *ngn-1* may have roles beyond the control of nervous system development.

### ngn-1 regulates expression of the paired-Like homeodomain transcription factor unc-42

To validate transcriptional targets downstream of *ngn-1*, we selected two transcription factor genes for further analysis, *unc-42* and *hlh-34*. Both genes are significantly down-regulated in the *ngn-1* transcriptome, suggesting that *ngn-1* has a role in activating their transcription.

The paired-like homeodomain transcription factor *unc-42* is orthologous to several mammalian genes including human Prop1 (Correa *et al.* 2019). *unc-42* positively regulates transcription of multiple genes including nuclear receptor 2E *fax-1*, which is also significantly down-regulated in our transcriptome (*P* = 0.009), and the glutamate receptor *glr-1* (Brockie *et al.* 2001; Wightman *et al.* 2005; Serrano-Saiz *et al.* 2013). Additionally, systems-level analyses of transcription factors suggest that *unc-42* may also act as a transcriptional repressor (Fuxman Bass *et al.* 2016). *unc-42* expression is significantly down-regulated in the *ngn-1* transcriptome (*P* = 0.006), suggesting that NGN-1 is required to activate transcription of this gene.

**Figure 7:**
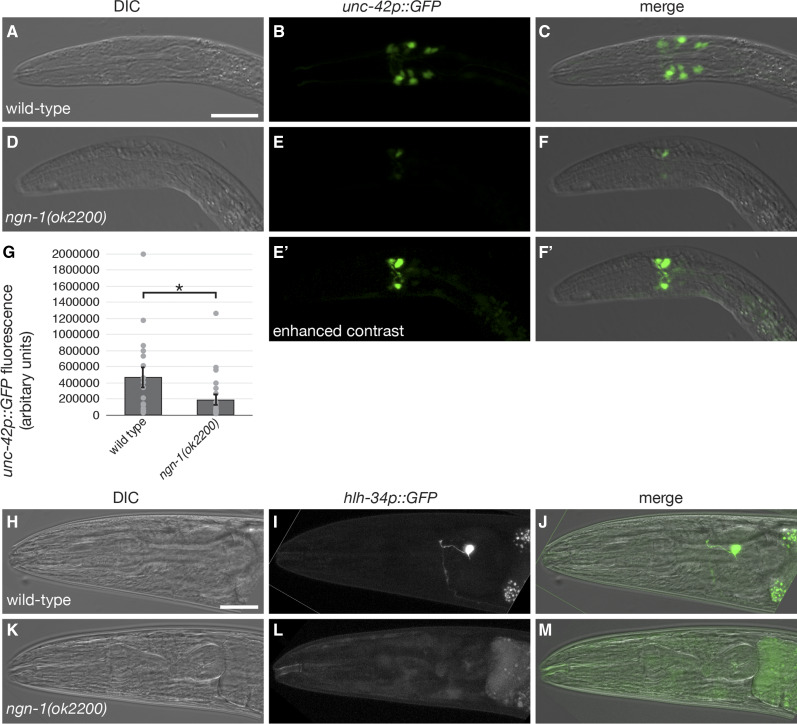
*unc-42* and *hlh-34* reporter gene expression is reduced or eliminated in *ngn-1**(**ok2200**)* mutants. (A – C) *unc-42p*::*GFP* expression in wild type and (D – F) *ngn-1* mutant L1 larvae. E’ and F’ show the same images as E and F but with contrast enhanced to show the number of cells expressing GFP. (G) Quantitative analysis of *unc-42p*::*GFP* expression in wild type and *ngn-1**(**ok2200**)* mutants (* *P* < 0.05, student’s *t*-test). Error bars show standard error of the mean and gray circles show individual data points. (H – J) *hlh-34p*::*GFP* expression in wild type and (K – M) *ngn-1* mutant young adults. Scale bar in panels A and H = 20 μm.

To validate our *unc-42* transcriptome result, we crossed an *unc-42p*::*GFP* transgene (Hope *et al.* 2004) into the *ngn-1**(**ok2200**)* mutant background and imaged L1 (first larval stage) larva via confocal microscopy. Wild type worms had an average of 13 *unc-42p*::*GFP* expressing head neurons (Figure 7A - C). When *unc-42p*::*GFP* expression was imaged in the *ngn-1**(**ok2200**)* background, GFP expression was significantly lower (*P* < 0.05) and restricted to less than 10 cells (Figure 7D – G). Figures 7E’ and 7F’ show the same images as Figure 7E and 7F but with the contrast enhanced to make the cells more visible. We conclude that NGN-1 has two roles in controlling *unc-42* expression. First, it controls the number of cells that express *unc-42*. Second, it controls *unc-42* expression levels by up-regulating *unc-42* transcription within those cells. Whether *ngn-1* is required for the actual fate specification of *unc-42*-positive cells is not known.

### ngn-1 regulates expression of the bHLH transcription factor hlh-34

We performed a similar GFP reporter gene analysis on *hlh-34*, which is predicted to code for a basic-helix-loop-helix transcription factor implicated in food-dependent behavioral adaptation via the AVJ interneurons, and is significantly down-regulated in our transcriptome (McKay *et al.* 2004; Grove *et al.* 2009; Lemieux *et al.* 2015). In concurrence with previous work, we confirmed that *hlh-34p*::*GFP* was expressed exclusively in the AVJ interneurons (Figure 7H – J). In *ngn-1**(**ok2200**)* mutants, *hlh-34p*::*GFP* expression was strongly suppressed, with only 5% of animals showing any GFP expression (Figure 7K – M; 19/386 animals scored, *P* < 0.001). To confirm and validate the identity of the *ngn-1**(**ok2200**)*; *hlh-34p*::*GFP* strain, we crossed hermaphrodites with wild type males and scored F1 male progeny for the presence of GFP. All F1 males (anticipated genotype *ngn-1**(**ok2200**)/+*; *hlh-34p*::*GFP/+*) were GFP-positive, confirming that our original strain was indeed homozygous at both the *ngn-1**(**ok2200**)* and *hlh-34p*::*GFP* loci. We conclude that *ngn-1* is almost exclusively required for *hlh-34* transcription during development, but that there is a low-level, partial redundancy in the transcriptional activation of *hlh-34* that can stochastically activate transcription in around 5% of animals.

## Discussion

### ngn-1 has multiple roles in C. elegans development

**Figure 8:**
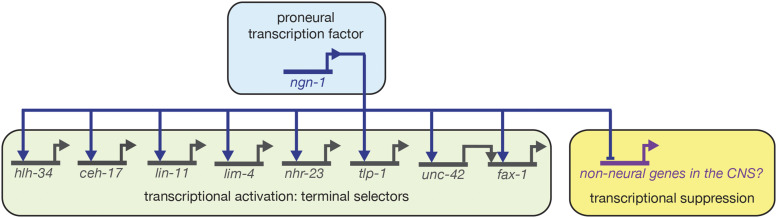
Summary of *ngn-1* transcriptional targets during embryogenesis as identified by RNAseq. While we have illustrated *ngn-1*’s activation of terminal selector transcription factors as direct, it is possible that additional factors act between *ngn-1* and the terminal effectors in this transcriptional cascade. The interaction between *unc-42* and *fax-1* summarizes previous work by Wightman *et al.* (2005).

This study sought to characterize roles for the predicted proneural transcription factor *ngn-1* in neural development and to identify downstream transcriptional targets using candidate gene and comparative transcriptomic approaches. Our data demonstrate roles for *ngn-1* in embryonic epithelial integrity, fate specification, cell body positioning, and overall morphology of multiple neuron classes (Figures 3 and 4). These data expand on previous work that identified a role for *ngn-1* in defining MI pharyngeal motorneuron fate (Nakano *et al.* 2010). The pleiotropic nature of *ngn-1* mutant phenotypes suggests roles for this gene in neuronal cell migration/location and architecture of key neurological structures such as the nerve ring. Surprisingly, we found almost no genetic interactions with known neuroblast migration and axon guidance genes, although the deep penetrance of *ngn-1* axon guidance phenotypes potentially masks any possible relationships (Table 1, Supplementary Figure 2). That being said, the transcriptional changes revealed in our *ngn-1* transcriptome point to a number of avenues that might explain some of the phenotypes observed. First, we identified eight transcription factors whose transcription are either directly or indirectly under NGN-1 control. These data were confirmed for both *unc-42* and *hlh-34*, whose expression is strongly suppressed in *ngn-1* mutants suggesting that NGN-1 is required for their transcriptional activation (Figure 7). By correlation, it is highly likely that *lim-4*, *fax-1*, *lin-11* *ceh-17*, *tlp-1*, and *nhr-23* transcription are also under direct or indirect NGN-1 control, suggesting that NGN-1 controls, in part, a cascade of at least eight downstream transcriptional regulators (Figure 8).

### NGN-1 has a role in nerve ring assembly

Our *kal-1p*::*GFP* reporter gene data shows that *ngn-1* has a role in directing nerve ring formation (Figure 3). This structure is initiated when SIA and SIB axons, along with the CEPshV glia, cluster together to form a process bundle that pioneers formation of the nerve ring (Kennerdell *et al.* 2009; Rapti *et al.* 2017). Follower neurons enter this track and reinforce its structure, with UNC-6/Netrin expression in CEPshV required for nerve ring axon guidance. *kal-1p*::*GFP* labels at least four cells required for nerve ring formation (SIAVL/R and SIBVL/R), making it a useful reporter for nerve ring structure (Supplemental Figure 2; Supplemental Table 2; Bülow *et al.* 2002). LIM-4 is expressed in a small number of cells including the SIA neurons (Sagasti *et al.* 1999). Our transcriptome data show that *lim-4* expression is controlled, in part, by *ngn-1* (Supplemental Table 3; Figure 8). In addition, SIA neuron identity is strongly correlated with genes down-regulated in our *ngn-1* transcriptome (Figure 5B). We speculate that *ngn-1* activation of *lim-4* transcription ultimately controls SIA fate, and/or expression of guidance cues expressed in SIA neurons, which helps establish nerve ring assembly.

### The role of ngn-1 in transcriptional regulatory cascades

In addition to its role in establishing nerve ring architecture, we show that *ngn-1* has roles in AIY interneuron axon guidance. However, NGN-1 likely has broader roles in interneuron specification and axon navigation. As mentioned above, both *unc-42* and *fax-1* transcription are down-regulated in *ngn-1* mutants (Figure 7). Previous work demonstrated that *unc-42* controls *fax-1* expression in some contexts but works in parallel with it in others (Much *et al.* 2000; Wightman *et al.* 2005). *fax-1* is required for expression of the NMDA receptor subunits *nmr-1* and *nmr-2* in the AVA and AVE interneurons, for *opt-3* expression in the AVE neurons, and *flp-1* and *ncs-2* expression in the AVK neurons. This suggests that NGN-1 sets up a regulatory cascade of transcription factors that control aspects of AVA and AVE interneuron fate and function. Also, FAX-1 is expressed throughout the life of the animal, suggesting that it functions as a terminal selector gene to maintain aspects of terminal fate and function in these neuron sub-types (Wightman *et al.* 2005; Hobert 2016). As such, this places NGN-1 close to the head of a neurodevelopmental cascade, activating expression of an intermediate factor followed by a terminal selector, which can subsequently maintain its own expression (Figure 8).

### The role of transcriptional repression in embryonic development

One of the surprising discoveries from our transcriptome study was NGN-1’s extensive role as a transcriptional repressor (Table 3; Supplemental Tables 3 and 4). Of the almost 500 genes whose transcription is significantly altered in *ngn-1* mutants, over 70% of these were up-regulated, indicating that NGN-1 normally functions to repress such transcription. Single-cell transcriptomics show that NGN-1 is expressed in both the AB and MS lineages at the 16-cell stage (Tintori *et al.* 2016). These data are confirmed by our *ngn-1p*::*GFP* time-lapse imaging (Figure 6). Whether NGN-1 represses the transcription of non-neural genes in neural lineages such as those derived from the AB founder cells, or neural genes in primarily non-neural lineages such as those derived from the MS cells (or a combination of the two) is not known. Work is ongoing to identify early acting GFP reporter genes that can be used to investigate NGN-1 transcriptional activation *vs.* repression at the single cell level during early embryogenesis.

### Transcriptomes as a way to investigate transcription factor function

Transcription factors and gene regulatory networks have been investigated in *C. elegans* using multiple techniques including genetic approaches (both forward and reverse), yeast-1 and 2-hybrid assays, chromatin immunoprecipitation, and protein binding microarrays (Grove *et al.* 2009; Walhout 2011; Gerstein *et al.* 2010; Fuxman-Bass *et al.* 2016; Hobert 2016). While these approaches have significantly illuminated how gene transcription is regulated during development and function, comparative transcriptomes provide a complementary approach to the above. Like forward genetic screens, transcriptomes have the advantage of being unbiased, in as much as they provide whole genome readout of transcriptional changes (Murray *et al.* 2012; Tamayo *et al.* 2013; Tintori *et al.* 2016). In addition, transcript changes identified in transcription factor RNASeq assays can illuminate downstream transcriptional activation *vs.* repression mechanisms. While systems-level approaches offer high-throughput combinatorial data on transcription factor interactions at the DNA and protein level, they are only as complete as the sets of genes or promoters/enhancers under investigation. For instance, NGN-1’s interactions with other bHLH transcription factors has been investigated using yeast-2-hybrid and immunoprecipitation approaches, which identified HLH-2 as the most likely binding partner to NGN-1 (Grove *et al.* 2009; Nakano *et al.* 2010). However, it did not reveal the preferred NGN-1 binding site nor its transcriptional targets. Our transcriptome cuts around this by identifying gene transcription changes in the *ngn-1* mutant background when compared to wild type. While this does not identify whether this is a direct or indirect interaction with a gene’s promoter, it provides a starting point for further investigation. Despite our transcriptome being based on RNA harvested from mixed-stage embryos (equating to around 12 hr of development), we were still able to identify over 500 targets whose transcript levels changed significantly. In particular, we identified *hlh-34* as a *ngn-1* target (Figure 7). Although *hlh-34* is only expressed in a single pair of cells (the AVJL/R interneurons), we still had the statistical resolution to identify this *ngn-1* target. Overall, this suggests that comparative transcriptomics offer a powerful general approach to identifying transcription factor targets during embryogenesis. Future work will refine our transcriptomics approach by taking this to a single cell or single embryo level to provide tighter analysis of either cell lineage or developmental timing.
